# Seminal Vesiculitis: A Rare Cause of Unilateral Ureteric Obstruction

**DOI:** 10.7759/cureus.73796

**Published:** 2024-11-16

**Authors:** Melissa Carvalho, David Curry

**Affiliations:** 1 Urology, Belfast Health and Social Care Trust, Belfast, GBR

**Keywords:** hydronephrosis, rare causes of ureteric obstruction, seminal vesiculitis, upper tract urothelial carcinoma, ureteric obstruction

## Abstract

The seminal vesicles are an accessory structure of the male reproductive system. The most common pathology associated with the seminal vesicles is infective, and patients may present with haematospermia, pain, and subfertility. Patients presenting with unilateral ureteric obstruction secondary to seminal vesiculitis are rare, and there are very few reported cases in the literature. This case report aims to review the presentation and management of such a case. A 59-year-old male presented to the emergency department with right-sided abdominal pain, vomiting, haematuria, and reduced urinary output. Blood tests showed raised inflammatory markers, hyperkalaemia, and a significant acute kidney injury with a creatinine of 695 µmol/L and an estimated glomerular filtration rate (eGFR) of 7 from a normal baseline. Non-contrast computed tomography (NCCT) imaging of the renal tracts identified an atrophic left kidney and a large soft tissue lesion at the level of the distal third of the right ureter, concerning for a primary ureteric malignancy. Notably, urine samples sent for cytology were reported as negative for malignancy. Following drainage and recovery from the acute episode, a timely outpatient ureteroscopy revealed no abnormalities of the ureter, and a subsequent magnetic resonance imaging (MRI) concluded right-sided seminal vesiculitis as the cause of this patient’s presentation. This case report demonstrates seminal vesiculitis as a rare cause of ureteric obstruction. It can mimic upper tract urothelial carcinoma (UTUC) and highlights the importance of a definitive diagnosis in patients with suspected upper renal tract transitional cell carcinoma.

## Introduction

We report an unusual case of unilateral hydronephrosis and renal impairment in the setting of a solitary functioning kidney and seminal vesiculitis. There are very few cases that have been reported in the literature, which will be examined in more detail within the discussion section.

The seminal vesicles are an accessory structure of the male reproductive system, responsible for secreting fluid that makes up the majority of ejaculate [1]. They are located on the posterolateral aspect of the bladder, between the bladder and rectum, and form a “V” shape. Inferior to the seminal vesicles lies the superior surface of the prostate [1]. The ureters are located superior and anterior to the seminal vesicles and pass posterior to the vas deferens [2,3]. Their close proximity explains why pathology in the seminal vesicles can affect the ureter, such as in this case.

Seminal vesiculitis is the term used to describe inflammation of the seminal vesicles and is most commonly infective in nature. It is often associated with concurrent infection elsewhere in the male genital tract, for example, prostatitis or epididymitis [4-6], and it is unknown whether it presents in isolation. It can be acute or chronic in nature. Other, less common pathology includes primary neoplasms of the seminal vesicles, which are very rare. Malignancy within the seminal vesicles is more commonly secondary to the local spread of advanced prostate, rectal, or bladder cancer. There are reported cases of primary seminal vesicle adenocarcinoma; however, this remains very rare, with fewer than 100 cases reported in the literature [7,8].

This case highlights the importance of consideration of non-malignant diagnoses mimicking upper tract urothelial carcinoma (UTUC), and using a structured approach to diagnosis.

This article was previously presented as an abstract and poster at “Research for Clinicians 2023,” on November 9, 2023.

## Case presentation

A male in his 50s presented to the Emergency Department with right-sided colicky abdominal pain, vomiting, haematuria, and reduced urinary output. He was normally well, with a past medical history of gout, hypertension, and polyarthritis. There was no history of sexually transmitted infections. He was a non-smoker and did not drink alcohol. His vital observations were stable on admission. On examination, he was tender in the right flank; however, there was no guarding or peritonism. A digital rectal examination revealed no obvious abnormalities. Blood testing on admission showed a normal white cell count but a significantly raised C-reactive protein, urea, creatinine, and potassium (Table 1). No recent baseline bloods were available. A urine screen was positive for leucocytosis and nitrites; however, cultures revealed no significant growth. Blood cultures were also negative.

**Table 1:** Laboratory investigations on admission eGFR, estimated glomerular filtration rate

Blood marker	Patient’s result	Reference range
C-reactive protein (mg/L)	336	<5
Serum urea (mmol/L)	30.5	2.5-7.8
Serum creatinine (µmol/L)	695	59-104
eGFR (mL/min/1.73 m^2^)	7	>60
Serum potassium (mmol/L)	6.4	3.5-5.3

**Figure 1:**
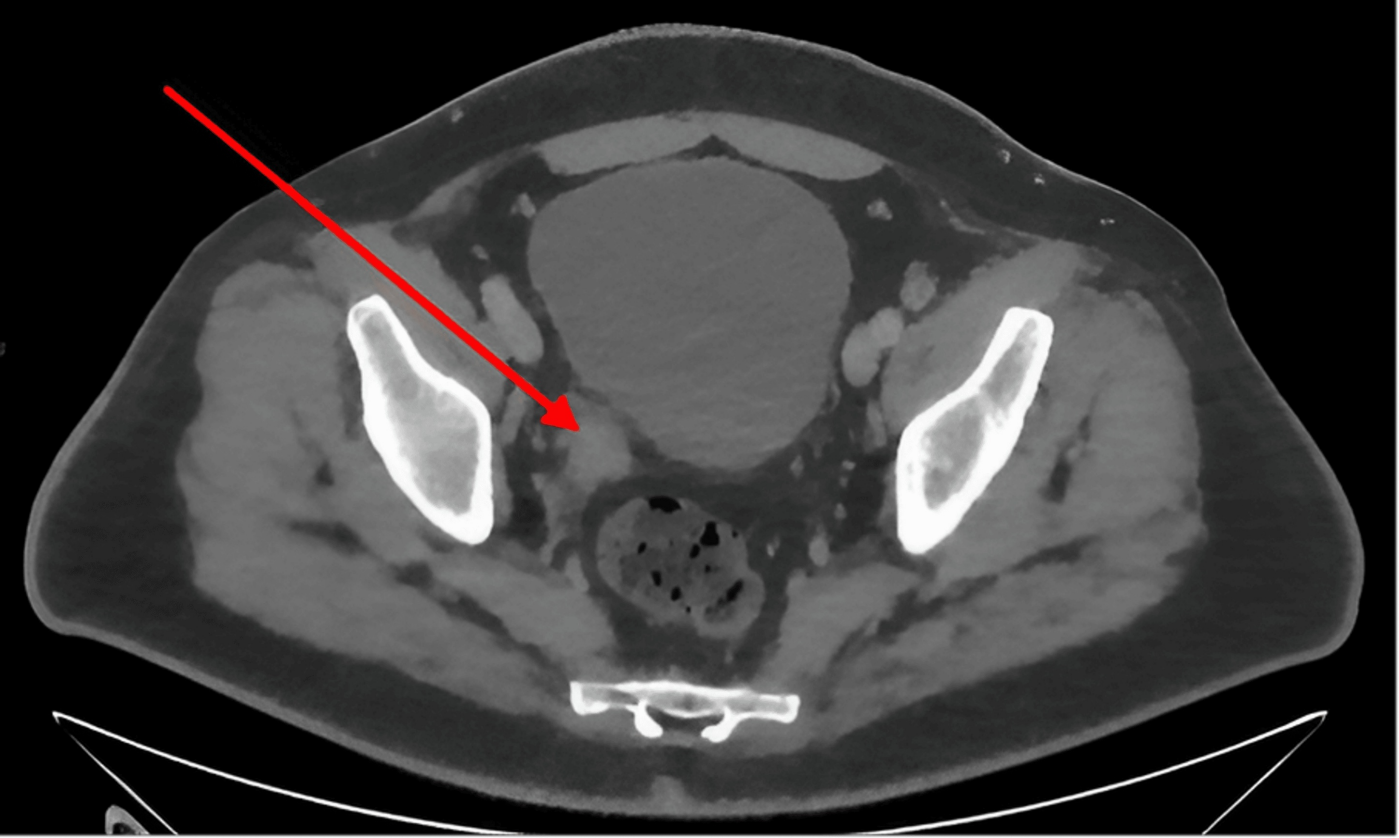
Cross-sectional CT image showing soft tissue mass at distal right ureter CT, computed tomography

The patient had a urethral catheter inserted and was commenced on broad-spectrum intravenous antibiotics. Non-contrast computed tomography (NCCT) of the urinary tract, completed soon after admission, showed a large soft tissue lesion at the level of the distal third of the right ureter, measuring 2.6 cm by 2.4 cm by 2.8 cm, resulting in moderate hydroureteronephrosis and concerning for a UTUC (Figure 1). Left renal atrophy was noted, and therefore, urgent decompression of the right kidney was undertaken.

**Figure 2:**
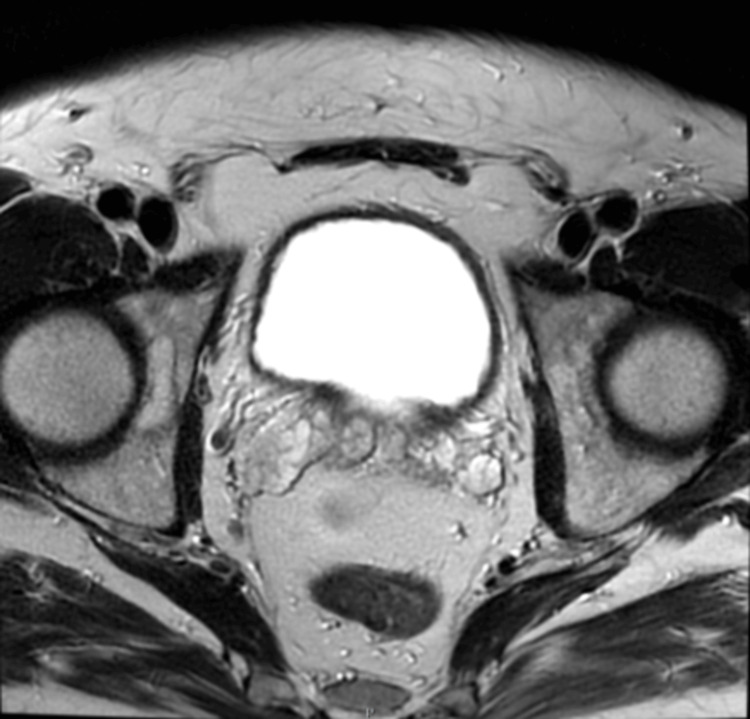
MRI image (axial T2) showing asymmetry of the seminal vesicles and an enlarged right seminal vesicle at level of vesico-ureteric junction MRI, magnetic resonance imaging

**Figure 3:**
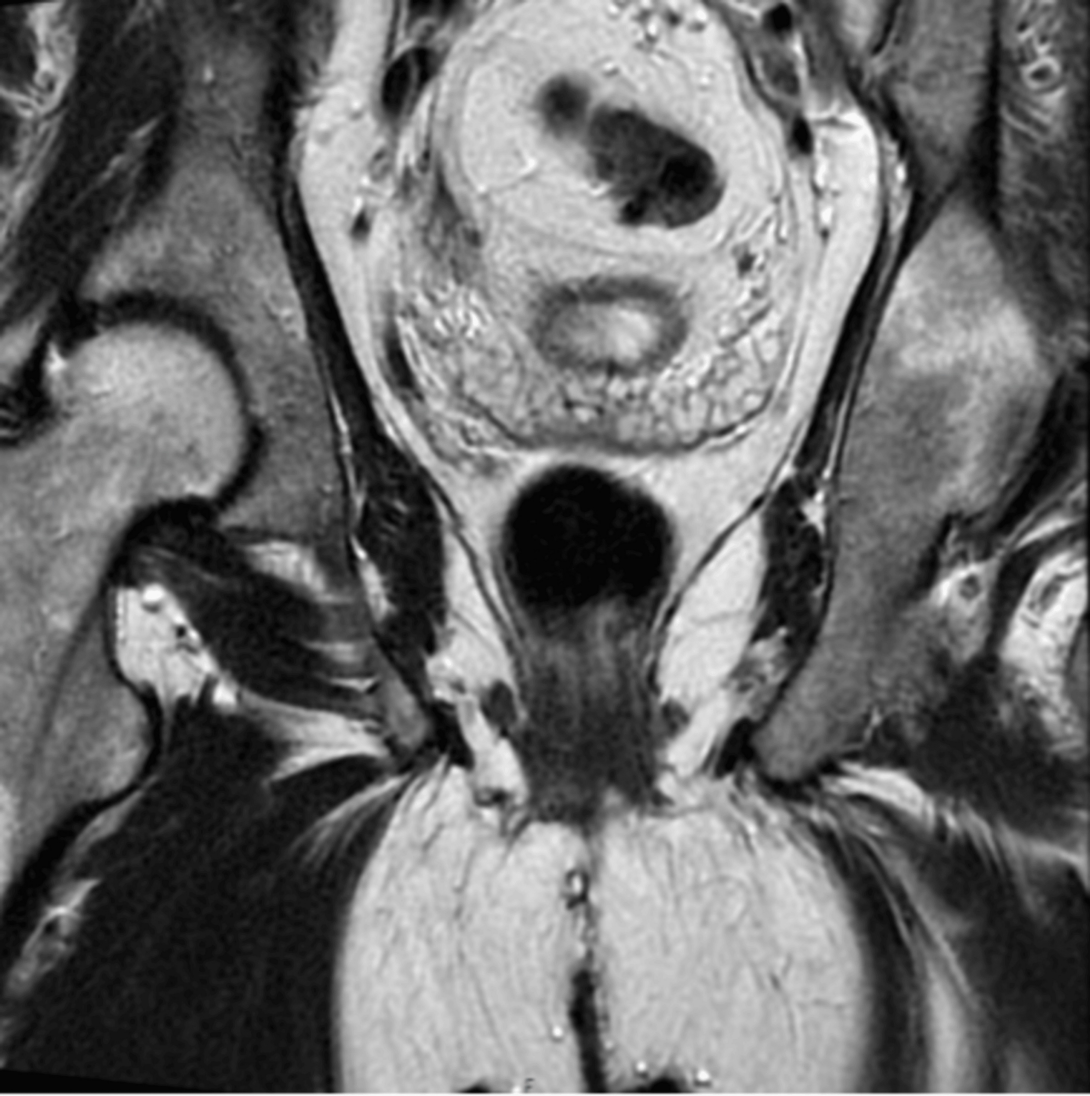
MRI image (coronal T2) showing enlarged right seminal vesicle with evidence of inflammation MRI, magnetic resonance imaging

Given the above findings, urgent drainage of the right kidney was achieved via the insertion of a nephrostomy tube, rather than a ureteric stent, owing to the urgency of the case and the availability of operating theatres and interventional radiology. As a result, the patient’s renal function steadily improved. An interval CT urogram and thorax scan showed a reduction in hydroureteronephrosis of the right renal collecting system and a persisting obstructing mass at the distal right ureter, with perinephric inflammatory change. There was no evidence of metastatic disease. With a working diagnosis of UTUC, the case was discussed at the uro-oncology multi-disciplinary meeting (MDM), which recommended urine cytology. The plan was to proceed to the right distal ureterectomy if the result was positive, and ureteroscopy if negative. Three urine samples from the nephrostomy tube were sent for cytology, all of which came back negative for malignancy. Renal function took a number of days to recover; creatinine halved from 670 µmol/L to 325 µmol/L in five days and was back to baseline (120 µmol/L) in one month's time. C-reactive protein normalised in two weeks. An outpatient cystoscopy and ureteroscopy revealed no abnormalities. Following this, a magnetic resonance imaging (MRI) of the abdomen (Figures 2-3) was completed, which revealed asymmetry of the right seminal vesicle with hyperintensity on diffusion-weighted imaging. It also reported that no solid mass lesion was associated with the right distal ureter, and there was no evidence of metastatic disease. A renal dimercaptosuccinic acid (DMSA) scan showed a left atrophic kidney with differential uptake of 15%, and 85% in the right kidney.

MRI appearances were thought to be in keeping with seminal vesiculitis. Consideration of a malignant process extrinsic to the ureter was given, but it was considered less likely. Further discussion at the MDM recommended a surveillance MRI, which was performed after six months. This showed no progression, but also no significant change in the soft tissue lesion, which measured 1.2 cm in axial length.

A nephrostogram showed free drainage to the bladder and no evidence of obstruction. The nephrostomy tube was removed after six months, and no recurrence of symptoms was seen. At 10 months follow-up, an ultrasound scan showed the kidney remained non-hydronephrotic, and creatinine was stable at 120 µmol/L.

## Discussion

Patients with seminal vesiculitis typically present with haematospermia, painful ejaculation, pain in the abdominal or lumbosacral region, and subfertility [7,9]. They can also have lower urinary symptoms, such as frequency, urgency, and dysuria [10]. In the above case report, the patient presented with abdominal pain, vomiting, haematuria, and raised inflammatory markers, which would be suggestive of infection. However, reduced urinary output and significant acute kidney injury are less typical presentations of seminal vesiculitis in isolation. Seminal vesiculitis is usually diagnosed with transrectal ultrasound, CT, or MRI [3,11]. Typical imaging findings include diffuse wall thickening. Interestingly, in this case report, CT imaging showed a soft tissue lesion at the level of the distal ureter but failed to identify this as seminal vesiculitis. MRI, however, was accurate in identifying enlargement and asymmetry of the seminal vesicle. Clinicians may often overlook seminal vesiculitis as a potential diagnosis due to its infrequent occurrence; however, the digital rectal examination will most likely reveal a distended and tender seminal vesicle, indicating this condition, as reported by Pugh in every case of his case series [12]. Management of acute seminal vesiculitis typically involves empirical antibiotic therapy, as the patient in this case report also received. Recurrent and persistent infections can increase the risk of abscess formation, where surgical drainage might be required [13]; however, this is a rare finding [6]. The literature has reported a limited number of cases where seminal vesiculitis led to ureteric obstruction. The earliest reports are from Mark and Hoffman, who describe three cases in 1922 [14]. Pugh describes a series of six case reports in 1928 and surmises that ureteric obstruction secondary to seminal vesiculitis is much more common than initially thought, and may present with incomplete or complete ureteric obstruction [12]. In both of the above case series, management involved diathermy to the seminal vesicles, serial cystoscopies with attempts to pass a ureteric catheter, and nephrotomy. Another report describes the infection progressing to a seminal vesicle abscess, resulting in ureteric obstruction and hydronephrosis [10]. In cases of hydronephrosis and hydroureter, the most likely cause is renal and ureteric calculi, followed by malignancy. In this case report, imaging excluded the presence of renal tract calculi; however, there were concerning findings suggestive of UTUC. For patients experiencing renal colic, clinicians should not dismiss the seminal vesicles as a potential cause and should consider performing a rectal examination. However, digital rectal examination may not accurately reflect underlying seminal vesicle pathology, as the limited reach of the examining finger and a distended bladder can make palpating the seminal vesicles challenging [7].

## Conclusions

This case report presents a rare instance of seminal vesiculitis leading to unilateral ureteric obstruction, a condition that mimicked UTUC in its presentation. This underscores the challenges of diagnosing seminal vesiculitis, as it is a rare condition with symptoms that can overlap with other urological diseases. Diagnostic imaging and a structured approach to exclude malignancy proved crucial in this case, and MRI, rather than CT, provided the definitive diagnosis, suggesting its importance in complex cases of obstructive uropathy.

This case emphasises the need for clinicians to consider non-malignant cases of ureteric obstruction, particularly in those who present atypically, and to employ appropriate diagnostic techniques, such as MRI and rectal examination, to secure an accurate diagnosis. I believe this case highlights the importance of obtaining a definitive diagnosis in patients with suspected UTUC before proceeding to radical treatment.

## References

[1] Magi-Galluzzi C (2023). Anatomy and histology of seminal vesicles. Uropathology.

[2] McKay A, Odeluga N, Sharma S (2023). Anatomy, abdomen and pelvis, seminal vesicle. StatPearls [Internet].

[3] Kim B, Kawashima A, Ryu JA, Takahashi N, Hartman RP, King BF Jr (2009). Imaging of the seminal vesicle and vas deferens. Radiographics.

[4] Furuya R, Takahashi S, Furuya S, Kunishima Y, Takeyama K, Tsukamoto T (2004). Is seminal vesiculitis a discrete disease entity? Clinical and microbiological study of seminal vesiculitis in patients with acute epididymitis. J Urol.

[5] Furuya R, Takahashi S, Furuya S, Saitoh N, Ogura H, Kurimura Y, Tsukamoto T (2009). Is urethritis accompanied by seminal vesiculitis?. Int J Urol.

[6] Cotter F, Sathianathen N, Thevarajah G (2022). Seminal vesicle abscess: a case report and review of the literature. J Clin Urol.

[7] Dagur G, Warren K, Singh N (2016). Detecting diseases of neglected seminal vesicles using imaging modalities: a review of current literature. Int J Reprod Biomed.

[8] Bouassida K, Hmida W, Mestiri S (2015). Primary seminal vesicle adenocarcinoma presenting as acute urinary retention and hematuria: a case report. World J Nephrol Urol.

[9] Li YF, Liang PH, Sun ZY (2012). Imaging diagnosis, transurethral endoscopic observation, and management of 43 cases of persistent and refractory hematospermia. J Androl.

[10] Imperatore V, Creta M, Di Meo S, Buonopane R, Spirito L, Mirone V (2017). Seminal vesicle abscess causing unilateral hydroureteronephrosis: a case report. Arch Ital Urol Androl.

[11] Kang YS, Fishman EK, Kuhlman JE, Goldman SM (1989). Seminal vesicle abscesses: spectrum of computed tomographic findings. Urol Radiol.

[12] Pugh W (1928). Seminal vesiculitis: a cause of ureteral obstruction. JAMA.

[13] Reddy MN, Verma S (2014). Lesions of the seminal vesicles and their MRI characteristics. J Clin Imaging Sci.

[14] Mark EG, Hoffman RL (1922). Renal retention due to seminal vesiculitis: a report of three cases. J Urol.

